# Editorial

**Published:** 1844-07-27

**Authors:** 


### THE MEDICAL EXAMINER.

PHILADELPHIA, JULY 27, 1844.

### EDITORIAL COURTESY.

A spirited, and, we should think, rather undignified war, has been going on for some time past between the Lancet and Medical Times, two ably conducted weekly medical journals, published in London. Such things are to be deplored by all true friends of the profession. Whatever the parties themselves may think, wrangling is always injurious to those immediately engaged in it, and the body suffers with its members. Impartial criticism, expressed in proper language, ought to give offence to no one. Just reproof, and dignified reply, are also proper, whenever the manner and the means employed by an opponent are such as a gentleman may notice. But when men of education, members of a learned and noble profession, descend to the use of vulgar epithets and personal abuse, they have no right to expect that honourable members of the profession will condescend to participate with them in such conduct, even so far as to feel interested in the controversy, much less to approve of it.

Great as is the evil of such example, in the present instance, science is in danger of losing by it in another way. By the *Medical Times* of the 29th ult., we are informed that the proprietors of that Journal are threatened with a prosecution by those of the Lancet for a breach of copyright, in consequence of the publication in the former Journal of abstracts of some important papers published in the latter. If it is to be held unlawful, or dishonorable, for one Journal to copy from another, giving the proper credit, either entire articles or abstracts of them, it will greatly circumscribe the usefulness of such publications. By this all parties must be losers. No Journal can be permanently injured by the transfer of valuable matter from its columns to the pages of another, when it is properly acknowledged; on the contrary, it serves to attract attention more generally to it, and thus promote its circulation. It is only when the matter of one journal is taken without acknowledgment by another,—in other words,—*stolen*, that there is any just cause of complaint. And even then, when not too flagrant, a noble mind will often bear the injustice rather than expose the culprit. Of the abundance of the rich, a little may be spared to the cravings of the starveling. Sometimes, however, justice cries aloud and bids us spare not, and then it may be the duty of the impartial journalist to do for the public good, what the generous author declines to do for himself. We have some examples of this kind laid aside, which we may sometime find space and occasion to use. Not those reckless and insane instances in which one man prints and publishes as his own the entire papers of another, either "translated from the French," or in "the King's English," but of that sort in which, by a kind of transmutation, or sweating process, the gold is extracted without the *appearance* of theft.

There are some men of such clever minds that after reading over a valuable essay, by a re-arrangement of its materials, and the aid of a little invention, they can accomplish the most perfect metamorphosis—so change the features that even the parent can scarcely recognise his own offspring. Others again, endowed with better memory and less imagination, reproduce it partly in the original form—here a phrase and there a word; now an odd technicality and then an anachronism;—a sort of patch-work concern, in which the original woof is strangely overlaid with other party-coloured stuff—too thin however to conceal the fraud.

### ANNUAL ANNOUNCEMENT OF THE PHILADELPHIA MEDICAL ASSOCIATION, FOR THE SESSION OF 1845.

Most of the gentlemen constituting this Association, have been successfully engaged in teaching their several branches, the past and present summer. By the present publication, we are informed that they have added to their number, and now present a complete organization, as follows, viz:

Robert Bridges, M. D. on Chemistry.
J. M. Wallace, M. D. on Surgery.
J. M. Allen, M. D. on Anatomy.
F. G. Smith, M. D. on Physiology.
J. F. Meigs, M. D. on Obstetrics.
Francis West, M. D. on Mat. Med. and Therpeutics.
Alfred Stillé, M. D. on General Pathology and Practice of Medicine.

The regular course of lectures of the Association for the session of 1845, will commence early in April, and continue until the end of October, with the usual recess during the summer.

Dr. J. M. Wallace, No. 1 Monroe Place, is the Secretary.

